# An interdisciplinary framework to optimize the anticipation skills of high-level athletes using virtual reality

**DOI:** 10.3389/fspor.2024.1324016

**Published:** 2024-02-12

**Authors:** Gilles Montagne, Nicolas Mascret, Martin Bossard, Loïc Chomienne, Simon Ledouit, Guillaume Rao, Nicolas Tordi, Eulalie Verhulst, Richard Kulpa

**Affiliations:** ^1^ Aix-Marseille Univ, CNRS, ISM, Marseille, France; ^2^ Univ Rennes, Inria, M2S, Rennes, France; ^3^ PEPITE EA4267, (EPSI), University Bourgogne Franche-Comté, Besançon, France

**Keywords:** virtual reality, top level sport, interdisciplinary, anticipation skills, ecological-dynamics

## Abstract

The ambition of our contribution is to show how an interdisciplinary framework can pave the way for the deployment of innovative virtual reality training sessions to improve anticipation skills in top-level athletes. This improvement is so challenging that some authors say it is like “training for the impossible”. This framework, currently being implemented as part of a project to prepare athletes for the 2024 Olympic Games in Paris, based on the ecological-dynamics approach to expertise, is innovative in its interdisciplinary nature, but also and above all because it overcomes the limitations of more traditional training methods in the field designed to optimize anticipation skills in top-level athletes. The ambition is to tackle successive challenges ranging from the design of virtual partners and opponents to the deployment of training programs in virtual reality, while ensuring the acceptability and acceptance of such innovative virtual reality training protocols and measuring associated workloads.

## 1 Introduction

Improving anticipation skills in high-level athletes is so challenging under normal training conditions that some authors say it is like “training for the impossible” (1). In this contribution, we wish to demonstrate that this challenge can be achieved provided an interdisciplinary framework is implemented to design a new generation of virtual reality (VR) training tools. We would like to put the emphasis on highly time-constrained sports for which anticipation skills are of paramount importance. It is now clearly established that experts differ from non-experts in their ability to gather and use the appropriate information. They are therefore able to make the right decision at the right time, despite drastic time constraints (2, 3). Based on this observation, the central question that arises is how training should be organized for athletes, including experts, to optimize anticipation skills.

Although a lot of research (4) conducted in recent decades has brought to light the main principles that prevail when learning sport skills in general, the specific optimization of anticipation skills is still a very challenging issue. In addition to the fact that both perceptual-motor and decision-making processes that take place are task-specific, dynamically related to action, and moreover not always clearly identified, training carried out on field can hardly meet the specifications of rigorous anticipation training protocols. Not only can the information content of a given task and the time constraints not be controlled precisely on field, but also their complexity is difficult to modulate, and the feedback given to athletes usually lacks precision.

In recent years, the revolution of digital technologies in the field of sport has opened new perspectives from this point of view (5, 6). Virtual reality technologies, provide potentially very powerful tools which may exceed the limits previously described and allow a perfect control of the information available for the athlete. It is now possible to immerse athletes in interactive and realistic 3D digital environments including biologically realistic moving partners and/or opponents while carrying out experimentally controlled tasks requiring anticipation, and to provide relevant feedback when needed. It is reasonable to consider that now is the time to “train for the impossible” and we posit that the conceptual, methodological, and technical solutions are readily available.

The rise of digital technologies this last decade, and more precisely recent evolutions in the domain of VR, have led the world of sport to take a close interest in the added value associated with the use of VR technologies for the purpose of optimizing performance. The large number of empirical works, literature reviews, and meta-analyses focusing on the role of VR in analyzing and optimizing the processes underlying sports performance reflects this enthusiasm (7–11). Studies conducted to date have revealed that VR is a tool of choice for discriminating the anticipatory skills of experts and non-experts in various sports such as baseball (12), golf (13), handball (14), rugby (15), and soccer (16). Curiously, as previously mentioned by some authors (7, 17), using VR in a training perspective to transform and optimize the processes underlying anticipation skills is much rarer. Moreover, very recent technological developments in VR Head-Mounted Display (VR-HMD) allow some limitations to be overcome (18).

Since many technological locks are being lifted, it is now conceivable to bring VR-HMD into the training environments of athletes and to deploy VR training sessions as part of a program focused on optimizing anticipation skills. However, a number of issues both methodological and theoretical still remain to be addressed before the program can be useful for athletes and coaches. These issues range from the design of animated digital twins of either opponents or partners, to the elaboration of training protocols in VR which are theoretically grounded and complementary to traditional training sessions, passing through the assessment of both the acceptance by athletes and coaches of technological solutions and the new workloads related to the deployment of VR training sessions. Only a dedicated interdisciplinary framework is likely to provide answers to the above-mentioned issues.

## 2 The need for an interdisciplinary framework

### 2.1 The example of an ongoing project

In order to give more meaning to our proposals we have chosen, as the main thread of this article, to refer to a current project which aims precisely to optimize the anticipation skills of high-level athletes in the context of preparation for the Olympic Games. The basic idea of this project is to improve the anticipation skills of athletes by confronting them with the digital twins of their opponents and/or their partners in a perfectly controlled setting.

In the case of 4 × 100 m relay, the team performance is partly based on the ability of athletes to synchronize their displacements and therefore to initiate their race at the right time, before the partner arrives in the relay transmission area, despite the pressure exerted by the opponents. VR training protocols will therefore have the function of training each athlete to initiate their race at the appropriate time, with an avatar based on motion capture of real sprinters and whose race features are configurable. In addition, the user will be able to benefit from precise feedback related to the timing errors produced.

In the case of boxing, the idea is to propose training protocols in VR that allow the boxer to learn to extract the appropriate information on the opposing fighter which should enable him/her to anticipate attacks and adopt the appropriate parry and counterattack. As mentioned previously in the relay example, the behavior of the virtual opponent can be parametrized, according to the boxers' needs. The use of VR will also allow online recording of the evolution of performance.

### 2.2 The need for an interdisciplinary approach

**Figure 1:**
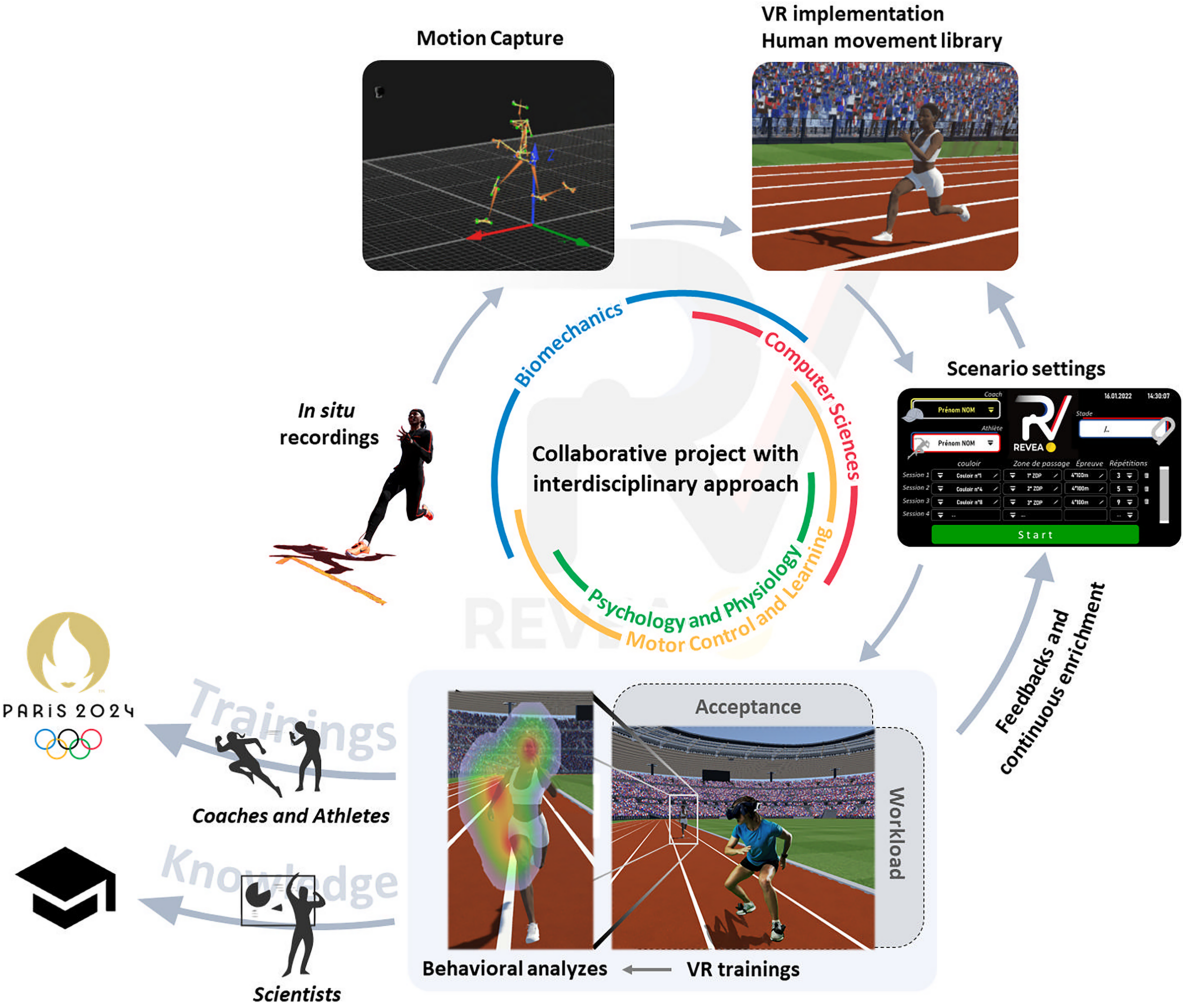
The objective of the proposed framework is to design virtual reality training sessions, used in a complementary way with traditional training methods, intended to optimize anticipation skills of top-level athletes. The basic idea is to pool complementary disciplinary skills from biomechanics, computer sciences, motor control and learning, psychology and physiology, to meet successive intertwined challenges. Firstly, *in situ* movement recordings will serve as a basis for the development of virtual character animations stored in an application intended to allow the end users (i.e., coaches and athletes) to schedule easily customizable training sessions. Secondly, the heart of the framework lies in the development of VR training methods most likely to optimize the underlying perceptual and decision-making processes, thanks to specific feedback accessible as needed to coaches and/or athletes. Finally, given our ambition to use VR training sessions in combination with traditional training sessions, both acceptance and workloads of VR sessions will be assessed as part of a risk mitigation strategy.

In the sport domain, the high level of performance attained by elite athletes is a perfect case for interdisciplinary research due to the multidimensional explanations of the determinants of their expertise (19, 20). But a recent review (21) asked the following question: *Is sports science answering the call for interdisciplinary research?* The authors concluded that this call is beginning to be fulfilled by sport science research, and they also highlighted that more individual analyses and more representative tasks are needed to really achieve this goal. The use of VR could meet these two expectations, but only if an interdisciplinary approach is conducted at the interface of life sciences, human and social sciences, engineering sciences, and computer sciences, mobilizing different scientific fields such as neurosciences, psychology, computer graphics, biomechanics, and physiology (Figure 1). Indeed, each scientific discipline contributes to first shed light on a particular field, for instance in the project mentioned in the previous section, motion captures (biomechanics), design of avatars with biological movements (computer graphics), training anticipation skills in VR (neurosciences), acceptance of the VR-HMD (psychology), and workload (physiology and psychology). But each disciplinary issue can positively or negatively influence the others (e.g., the biological movement of an athlete's avatar can, from a psychological point of view, be accepted or on the contrary be refused by the VR-HMD user). It is thus the interdisciplinary approach, at the interface of all these disciplines, that will ensure the success of the project.

## 3 Toward the optimization of anticipation skills

### 3.1 A prerequisite: a theoretical conception of the processes to optimize

Although an interdisciplinary consortium is mandatory to lift all the locks associated with the implementation of training protocols in VR designed to optimize anticipation skills in top athletes, it may not be sufficient to succeed. The whole project must be based on a clear conception of the anticipation processes implemented by athletes that one wishes to train and optimize. Distinct theoretical frameworks account for the processes underlying anticipation skills (22, 23). For example, the project mentioned previously (§ 2.1) is backed on the ecological-dynamics vision of expertise in sport (24). According to this theoretical framework, expertise relies on the “athlete's capacity to functionally adapt his/her movements to the dynamics of complex-performance environments by continuously perceiving information to regulate goal-directed actions” (24, p. 130). This framework has its origin in the work of Gibson (25) which proposed to break with the traditional cognitivist-inspired approaches to perception, by proposing a new ecological approach to perception and action based on the inseparable nature of the links between the agent and his/her environment and between perception and action (26). According to the ecological-dynamics framework, athletes would actively perceive opportunities for action in the perceptual flow produced by moving partners and/or opponents, provided they are attuned to the relevant high-order perceptual variable. The optimization of perceptual-motor and decision-making processes is a question of actively discovering these variables through repetitions in various contexts in the presence of feedback.

In the light of these considerations, VR training sessions should preserve the natural links between perception and action insofar as the anticipation skills of experts cannot be optimized when the natural links between perception and action are disrupted. Training sessions should also preserve as much as possible both the task and environment constraints encountered by the athlete on field in such a way as to facilitate the transfer of the optimized skills to the field (27). With these specifications in mind a research project can be designed to overcome intertwined challenges (Figure 1).

### 3.2 The main challenges

Challenge #1: Designing realistic partners’ and opponents' avatars immersed in representative environments.

The first challenge lies in the design of avatars of either partners and/or opponents that athletes will face during VR training sessions but also to integrate these avatars into representative virtual environments identically reproducing the sporting environments. This ambition requires the realization of a series of operations described in Figure 1. The first one concerns the collection of *in-situ* motion datasets representative of consistent motor performances with respect to the levels of expertise and individual movement patterns of the athletes. It is crucially important to carry out such motion capture in real field conditions while preserving as much as possible the biological accuracy of the collected movement pattern. The second step is to transfer these motion datasets into virtual character animation. The challenge is to apply these movements to fully reconfigurable avatars while preserving the biological properties of these motions. This requires morphological retargeting approaches.

The needs concerning the characteristics of the avatars (e.g., adaptive vs., non-adaptive) will depend on the sporting situation considered. Concerning the project described in section 2.1, the use of adaptive avatars is unnecessary in the context of the 4 × 100 m relay as virtual reality is only used to optimize the initiation of the race of the athlete who will receive the stick. Conversely, in boxing, the use of adaptive avatars is crucial to maintain the dynamics of the sport and ensure that the virtual boxer is at the right position and orientation for a successful attack. Its performance is also automatically adjusted to match the real boxer's capabilities, preventing it from being placed in a situation of failure and allowing an individual adaptation to the boxer's progress.

As a corollary to the previous point, the selection of athletes for motion captures can be important. For 4 × 100 m relay, which constitutes a collaborative task, it is logical for example that the motion captures are carried out on the partners. For boxing, the confrontation with opponents, whoever they may be, allows boxers to optimize their anticipation skills, especially when attacks are selected to mimic opponents' preferred ones.

Challenge #2: Designing a user-centric app.

The *in-situ* motion datasets previously mentioned will serve as the basis for creating a library of scenarios that will be used during the VR training sessions. For example, in the case of 4 × 100 m relay, these scenarios take the form of sprinters approaching the transmission zone, either alone or in the presence of opponents, at fully configurable speeds and places on the track. In the case of boxing, these scenarios immerse a real boxer, in a virtual ring, facing a virtual opponent who will produce a series of attacks that challenge the defensive skills to be trained. The scenario library mentioned previously can be stored in an application whose specifications has to be designed in close connection with end users. The basic idea is to allow each coach to easily program the VR training sessions in advance or at the last moment by selecting the appropriate scenarios. In return the application is intended to record a number of variables to characterize the athlete's behavior in order to be able to give feedback to the athlete and/or the coaches online or in a delayed way as required.

Challenge #3: Designing theoretically grounded VR training sessions.

The third challenge is to identify the best way to organize the training of athletes to optimize their anticipatory skills. The use of VR finds its full expression by allowing a great number of repetitions in a fully controlled environment and by providing precise feedback about the choices and actions made by the athletes. The ambition here is to create individualized training sessions allowing the athlete to develop adaptive behaviors; for example, the variability of the conditions of practice at the service of the development of perceptual-motor workspaces within which experts can search for functional coordination solutions (24). Importantly, VR allows the implementation of training methods that would not have been possible otherwise. The action capacities of either partners and opponents can be easily manipulated, so as approach biological limits or even exceed them, in order to develop athletes' adaptive capacities and prepare them as much as possible for the imponderables that characterize very high performance. Importantly, to preserve the ecological validity of training protocols, the sensory modalities generally used (in addition to the visual modality) must also be stimulated as part of Virtual Reality training. For example, in the case of the 4 × 100 m relay, stadium noise has been integrated to contextualize the training as much as possible. However, no haptic feedback is needed. For the boxing task, acoustic feedback is added on impacts, and spectators' noise can be set from hostile to friendly.

Challenge #4: Overcoming the limitations of VR.

VR is a hardware- and software-based system and therefore has some intrinsic limitations. First, it has been widely demonstrated that depth perception is underestimated in virtual environments (28). This problem can be overcome by immersing free-moving athletes in rich and structured virtual environments providing them with a large amount of motion parallax and other sources of information which is well documented to greatly attenuate virtual misperception (29). Secondly, VR can lead to cybersickness, a user discomfort that can result in discontinuation of use. Its main causes are hardware, content, and human factors (30). To avoid cybersickness, the type and duration of training sessions can be balanced based on the athlete's personal characteristics and habits with VR. Thirdly, the main challenge raised by the use of VR for training concerns skills transfer. Does improvement in VR entail improvement in real life? This is a very complex issue as performance is by nature multifactorial, and assessment must be carried out using protocols that are both close to competition conditions and quantifiable. Importantly, some recent work reveals that anticipation skills optimized through virtual reality training protocols can be effectively transferred to the field (8).

## 4 Two risk mitigation strategies

Two risk mitigation strategies have to be conducted simultaneously, namely evaluation of acceptance and workloads of VR sessions. The objective is to be sure that the benefits on the optimization of anticipation skills are not degraded by other factors that are not controlled.

### 4.1 Assessing acceptance of the VR-HMD

If the VR-HMD is not accepted by coaches and athletes before a first use, it would ultimately never be used. Therefore, based on the Technology Acceptance Model (TAM, 31), it is of paramount importance to identify which variables influence coaches' and athletes' acceptance of the VR-HMD and thus lead, before a first use, to initial blockages regarding its use for sports performance improvement. For example, in the project mentioned previously (see § 2.1) it has been shown (32) that elite athletes intended to use this VR-HMD, found it quite useful and quite easy and pleasant to use, even though they have not really used it yet. It also highlighted that elite athletes wonder if those around them would advise them to use it. Therefore, initial blockages do not seem to appear in elite athletes. The issue of acceptance is not limited to acceptance before first use. If the VR-HMD is not accepted once it has been tested, its effective use quickly declines. Therefore, it is necessary to regularly measure coaches' and athletes' acceptance of the VR-HMD to identify to what extent specific stages of software development led to changes in perceived usefulness, perceived ease of use, perceived enjoyment, and intention to use the VR-HMD. Finally, once the VR-HMD is in its final version and delivered to coaches and athletes, the same variables should be regularly measured throughout the training process to identify whether acceptance of the device still remains at a high level and whether specific interventions are ever needed if acceptance decreases.

### 4.2 Assessing workloads

Adding VR-based training protocols to the athletes' training program would change the total training load. The primary goal is indeed to allow athletes to train more without high-intensity work or risk of injury, but training may also add an attentional, cognitive, and physiological workload. Therefore, it is necessary to monitor and prevent any possible negative effect on the training load induced by using VR in training when the athlete is not accustomed to this technological device. An Athlete Management System can be used to collect and analyze data related to the training program and to the athletes' responses to this program. Different types of information related to exertion, fitness level, motivation as well as pain perceived, and sleep quality and duration can be collected and analyzed. Questionnaires are also a way of reporting subjective experience of workload. The simulation task load index (SIM-TLX, 33) was specifically designed for its self-reported assessment in virtual environments. Combined with the Athlete Management System and with external loads (e.g., heart rate), these measures will help in identifying potential overload but also in individually adapting and enhancing VR training (34). All these variables can be analyzed to examine how the athletes used VR. In this way it is possible to prevent all negative effects related to RV added to the training program and determine which type of use is the most beneficial.

## 5 Conclusion and perspectives

Despite the growing interest of the sports world in new technologies, and XR technologies in particular, they are little adopted by top coaches and top athletes as part of training procedures. This article aims to show how an interdisciplinary framework can pave the way for the deployment of innovative virtual reality training sessions to improve anticipation skills in top-level athletes. VR is particularly attractive because it offers the opportunity to overcome the limitations of traditional training protocols by allowing athletes to optimally develop their adaptive capacities. The DNA of this framework is based on its interdisciplinary nature, but also on a precise knowledge of the processes we wish to optimize which guides the technological choices and the use made of them. We believe that the approach could enable new generations of studies to be carried out to draw out the quintessence of XR tools in the context of optimizing the performance of high-level athletes.

## Data Availability

The original contributions presented in the study are included in the article/Supplementary Material, further inquiries can be directed to the corresponding author.
